# The effect of educational intervention based on the theory of planned behavior on improving physical and nutrition status of obese and overweight women

**DOI:** 10.1186/s12905-022-01593-5

**Published:** 2022-01-15

**Authors:** Ali Khani Jeihooni, Mehdi Layeghiasl, Asiyeh Yari, Tayebeh Rakhshani

**Affiliations:** 1 Nutrition Research Center, Department of Public Health, School of Health, Shiraz University of Medical Sciences, Shiraz, Iran; 2 Departement of Health Promotion, School of Health, Shiraz University of Medical Sciences, Shiraz, Iran; 3 Department of Health Education and Health Promotion, School of Health, Hormozgan University of Medical Sciences, Bandar Abbas, Iran

**Keywords:** Nutrition, Obesity, Overweight, Physical activity, Theory of planned behavior

## Abstract

**Background:**

Regarding the high rate of obesity and overweight among women, develop a comprehensive and effective program it seems necessary to improve their nutritional behaviors and physical activity. This study aims to survey the effect of educational intervention based on the theory of planned behavior (TPB) on improving physical and nutritional activities of obese and overweight women.

**Methods:**

This experimental study was performed on 400 obese and overweight women over the age of 20. The sampling method was A simple random sampling. The data collection was valid and reliable self-reports measure, questionnaires. This tools was including demographic information, questionnaire based on the constructs of the theory of planned behavior, physical activity performance questionnaire and nutritional performance questionnaire that individuals completed before and 6 months after the educational intervention. The training intervention for the experimental group consisted of 12 sessions of 50–55 min. Data analyzed by SPSS22 and by using chi-square test, independent t-test and paired t-test.

**Results:**

Findings showed that before the educational intervention, was no significant difference between the experimental and control groups in terms of education, household monthly income, occupation, mean age, marital status, awareness, attitude, perceived behavioral control, subjective norms, physical activity and nutritional behavioral intentions, and physical activity and nutritional performance, weight and BMI. However, six months after the training intervention, there was a significant increase in each of the TPB contracts, weight and BMI in the experimental group, while no significant difference was observed in the control group. The meaningful level was considered 0.05.

**Conclusion:**

Our findings partially support of applying theory of planned behavior in reducing the weight, BMI and improved nutritional performance and physical activity of the study subjects. TPB could be an important strategy for effective future educational interventions.

## Background

Non-communicable diseases (NCDs) account for 72.3% of all deaths around the world and are known as the leading cause of deaths worldwide [1]. About 82% of the deaths in Iran are also due to NCDs [2]. Common NCDs such as diabetes, cardiovascular disease, and cancer have a great impact on quality of life, performance, and social costs of individuals in terms of health and loss of productivity [3]. Given that more than half of premature adult deaths are due to unhealthy lifestyles [4, 5], diet and physical activity can be considered as the most important lifestyle behaviors to reduce the risk of NCDs [3]. Inactivity is the fourth leading cause of death in the world, accounting for approximately 30% of cardiovascular diseases, 27% of all types of diabetes, and 21–25% of breast and colon cancers [6]. In 2012, 38 million out of 56 million deaths worldwide were due to NCDs, one of the main risk factors for which was inadequate physical activity [7]. Therefore, inadequate physical activity is a significant risk factor for NCDs. However, 23% of adults over the age of 18 in the world do not have sufficient physical activity [8]. In Iran adults over the age of 18, about 32% of the population (22% of men and 42% of women) are considered inactive [2]. Overweight and obesity are also considered as the main risk factors for such diseases [9], and about 65% ​​of Iranian women are overweight or obese [10]. In addition, nutritional behaviors are known as the most preventable factors affecting NCDs and obesity [11, 12]. The results of studies in Iran show that the food basket of Iranian women is moving towards a decrease in quality. These changes may be due to inconsistencies in dietary patterns with people's awareness of a healthy diet. Also, individual and interpersonal factors, cultural factors, food interests and preferences of the individual and other family members are among the most important factors that affect the nutritional status of women [13]. But, this is also true about infectious diseases. For example, now that the world is suffering from the Covid-19 pandemic, proper nutritional behaviors and adequate physical activity can help strengthen the immune system to combat this disease [14]. Overweight and obese people are more prone to severe complications of Covid-19 and the death from it [15]. Thus, according to the results of most studies that showed obesity and overweight were more common among women than men and a small number of women had a healthy lifestyle, changes in their lifestyle may not only improve their own health status, but can also play an effective role in improving the lifestyle of other family members [16]. Women make up about half of the total population of Iran, and regarding the high rate of obesity and overweight among them, developing a comprehensive and effective program to improve nutritional behaviors and physical activity of this very large population group seems necessary. To promote women's health, educating them is of special importance and can be effective in improving their lifestyle as well [17]. In this regard, the existing theoretical frameworks for analyzing and changing behavior are a good guide for effective planning and intervention. Health promotion programs based on theories and models will lead to useful and effective results [18]. Human behavior is affected by various factors, the identification of which is essential for designing and implementing educational programs to improve individuals’ health. The first effective step in this process is to select an appropriate model or theory. One of the effective models used for the successful transformation of undesirable behavior into health behavior is the theory of planned behavior [19, 20], which considers the individual’s beliefs, social factors, and motivation to follow important people in life as a set of factors influencing behavior change.

The theory of planned behavior (TPB) was developed by Ajzen & Fishbein in 1980, and is one of the behavior change patterns (cognitive-social model of value expectation). As a theory of behavior change, it states that intention is the main determinant of behavior, and is influenced by the following three independent constructs: individual’s attitude toward behavior, subjective norms, perceived behavioral control [19, 20].

However, numerous successful studies have been conducted worldwide to promote physical activity and nutritional behaviors of different populations using this model [21–25]. Considering the importance of nutritional behaviors and physical activity in overweight and obese women, which comprise more than half of the Iranian female population, the present study aimed to determine the effectiveness of the TPB in education nutritional behavior and physical activity of overweight and obese Iranian women.

## Methods

This is an experimental study conducted on 400 obese or overweight women with a BMI of 25 ≤ under the auspices of the health centers in Fasa city, Fars province, Iran, from February to December 2019. From among the six urban health centers of Fasa, two were randomly selected for sampling (one center for the experimental group and one for the control group). A simple random sampling was done in each health center using the number of the women's household health records in the mentioned centers. The samples were then invited to attend a health center on a certain day to get acquainted with. They were also explained the study objectives, and their informed consent was obtained. The sample size was calculated to be 163 based on the study by Shakerinejad et al. [26] and using the formula for comparing the mean variables. Due to the possibility of falling study samples and lack of cooperation, 200 people were assigned to each group.

According to the women's health records, those with a BMI of 25 ≤ were included in the study. For higher accuracy, the women's heights and weights were measured once more and their BMIs were calculated. The inclusion criteria were as follows: BMI of 25 ≤ , waist circumference 80 cm or more, being 20 ≤ years old, not receiving psychotherapy or other regular weight loss and exercise program simultaneously, not taking psychotropic or weight-affecting drugs, lack of pregnancy, and lack of thyroid problems or diabetes. A German Beurer digital scale with an accuracy of 0.1 kg was used to weigh the women. To calculate BMI, all women were weighed with light clothing and no shoes, using the digital scale. Their heights were also measured using a caliper with an accuracy of 0.5 cm installed on the wall, while the women were standing tall without shoes so that the knees, hips, shoulders, back, and head were in the same direction. The caliper was applied tangentially on the scalp so that the hair was flat.

**Fig. 1:**
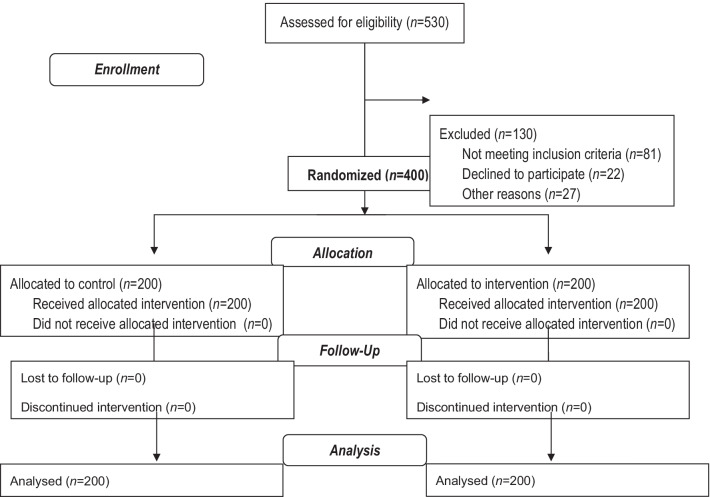
Flow chart of study

The exclusion criteria were specific diets due to cardiovascular diseases, hypertension, and diabetes, exercise program for weight control, unwillingness to participate in the study and being absent from the educational program for more than 2 sessions. Figure 1 presents the study flow diagram.

The data collection tool was based on Jeihooni et al. [27], Khayeri et al. [28], Didarloo et al. [29], Baji et al. [30], Cheng et al. [31] and Soorgi et al. [32] However, the validity and reliability of the questionnaires used have already been confirmed in these studies. This tool was including demographic information (age, education, occupation, marital status, family income, and household size), and a questionnaire developed based on the TPB. The latter included 20 multiple-choice questions on awareness (1 score for each right answer and 0 for each wrong answer, with a minimum total score of 0 and a maximum of 20), 15 questions on attitude using a 5-point Likert scale from strongly disagree (score 1) to strongly agree (score 5) with a minimum total score of 15 and a maximum of 75, 15 questions on perceived behavioral control on a 5-point Likert scale from strongly disagree (score 1) to strongly agree (score 5) with a minimum total score of 15 and a maximum of 75, 10 questions on subjective norms on a 5-point Likert scale from strongly disagree (score 1) to strongly agree (score 5) with a minimum total score of 10 and a maximum of 50, 10 questions on physical activity. Intention on a 5-point Likert scale from strongly disagree to strongly agree (scores 1 to 5) with a minimum total score of 10 and a maximum of 50, and 10 questions on nutritional practice intention on a 5-point Likert scale from strongly disagree to strongly agree (scores 1 to 5) with a minimum total score of 10 and a maximum of 50.

The physical activity performance questionnaire which was used in the study of Khani Jeihooni et al. [27] consisted of 20 questions on duration and type of physical activity on each day of the week, and the individual’s performance was given a score of 0 to 20. The nutritional performance questionnaire consisted of 20 questions on the type and amount of food consumed during the past week, with the minimum and maximum scores of 0 and 20, respectively. The individuals' performance was recorded based on their activities and self-reports.

After selecting the experimental and control groups, the research objective and process were explained to each of the women as well as the health centers staff. The questionnaire was completed by the experimental and control groups and the women’s weights were measured.

Considering the pre-test results, the educational content was prepared based on the TPB. The educational intervention for the experimental group consisted of twelve 50-to-55-min educational sessions including lectures, questions and answers, group discussions, use of educational posters and pamphlets, presenting films and PowerPoint slides. The educational program was provided by a health education and health promotion PhD holder, a nutritionist, and a psychologist collaborated with two experts in women's health and NCDs in Fasa health center (Table 1). The members of the intervention group were divided into 5 subgroups of 40 people and participated in training sessions twice a week in the hall of the health center.

**Table 1:** Content of training sessions based on the theory of planned behavior

Session/concept	Educational content	Educational method
First	Explaining the effects and complications of obesity and overweight, the importance of preventing chronic diseases, and encouraging the use of a healthy diet, and physical activity and also discussed about them	Brainstorming thinking، and discuss the positive consequences
Knowledge and attitude		
Second	Provide statistics on the complications of obesity، Provide facts about obesity and not doing exercise and the problems caused by them	Lectures, preparation of fact sheets
Knowledge and attitude		
Third and fourth	Discussing and Examining beliefs individual and social, and Normative social groups about the physiological-psychosocial effects of obesity, It was held in the presence of women's wives or a family member and officials of health centers	Includes role-playing and psychological play and panel discussion and videoclips
Subjective Norms		
Fifth and sixth	Discuss identifying negative moods and replacing them with rational and positive thoughts, helping people identify barriers to behavior and overcoming barriers. A 41-year-old woman who was able to lose weight with physical activity and proper nutrition was invited to talk about obesity and its problems and how to lose weight for people	Discussion of facilitators and incentives and breaking behavior into small steps and reducing inhibitors and stress caused by behavior change and videoclips
Perceived		
Behavioral control		
Seventh and eighth	Discuss and help people identify high-risk situations and improve coping skills with high-risk situations, discuss the benefits of healthy eating and exercise and its positive effects on health, discuss the harms of sedentary lifestyles and unhealthy eating	Lectures, questions and answers, group discussion, videoclips and PowerPoint
Intention		
Ninth and tenth	Weaknesses, fears, communication problems, positive and negative experiences were identified and discussed Provide opportunities for appropriate encouragement and collective feedback	lectures, questions and answers, group discussion, videoclips and PowerPoint
Behavior		
Eleventh	Family walks and group sports in the park	Lectures, questions and answers, group discussion, videoclips and PowerPoint
	Walking, running, rhythmic movements	
	Teaching how and steps to do physical activity, warming up, main activity and cooling down	
Twelfth	Summarizing and reviewing training sessions and presenting and expressing people's experiences	Lectures, questions and answers, group discussion, videoclips and PowerPoint

At the end of the meetings, each participant was given a booklet and an educational CD. The women Intervention Group were divided into groups of 10–15 and formed groups of friends and partners. In order to maintain and promote the activities of the experimental group members, they received an educational text message per week. A WhatsApp group was also created to exchange information and the individuals were asked to record their nutritional and physical activities in the specified forms. Physical activity training was in three parts: warm-up, exercise, cooling, and included sports such as walking, stretching, and rope for 30–45 min. Based on the time they did physical activity during the week and recorded in the form and also evaluated by a questionnaire. One month and three months after the educational intervention, two follow-up sessions were held to examine the behavior of women by viewing the booklets in which they recorded their activities.

Six months after the intervention, both the experimental and control groups completed the questionnaire, and weights were measured again. At the end of the study, an educational session was held for the control group and they were given an educational booklet each. To observe ethical considerations, not only the permission was obtained from the ethics committee of Fasa University of Medical Sciences and Fasa Health Center, but the women were justified about the study, and their informed consent was obtained. In addition, the objectives, importance, and necessity of conducting the research project were explained to them, and they were ensured that their information would remain confidential. The data were analyzed using the SPSS 22 software as well as the chi-square test, independent t-test, and paired t-test. The significance level was considered 0.05.

## Results

The participants in this study were 400 women over the age of 20. There was no significant difference between the mean age and mean family size in the intervention and control groups based on independent t-test. Chi-square test showed that there was no statistically significant difference between the two groups of test and control in terms of education, monthly household income, job and marital status (Table 2).

**Table 2:** Comparison of frequency distribution of demographic variables in the experimental and control groups

Variable	Experimental group		Control group		P-value
	N = 200		N = 200		
	Number	Percent	Number	Percent	
Occupation					
Housewife	172	86	163	81.5	0.193
Employed	28	14	37	18.5	
Monthly household income					
< 30 million rials	112	56	102	51	0.184
30–60 million rials	58	29	65	32.5	
> 60 million rials	30	15	33	16.5	
Education level					
Primary	23	11.5	20	10	0.179
Secondary	38	19	42	21	
High school	97	48.5	98	49	
Higher education	42	21	40	20	
Marital status					
Single	14	7	12	6	
Married	168	84	172	86	0.241
Divorced	8	4	10	5	
Widow	10	5	6	3	

Chi-square test

The results showed that before the educational intervention, there was no significant difference between the experimental and control groups in terms of awareness, attitude, perceived behavioral control, subjective norms, physical activity and nutritional behavioral intentions, and physical activity and nutritional performance. However, a remarkable increase in each abovementioned variable in the experimental group six months after the educational intervention indicated a significant difference, while no significant difference was observed in the control group (Table 3).

**Table 3:** Comparison of mean scores of awareness, attitude, perceived behavioral control, subjective norms, behavioral intention, physical activity performance, and nutritional performance of the women in the experimental and control groups before and six months after the educational intervention

Variable	Group	Pre-intervention M ± SD	Six months after intervention M ± SD	p-value
Awareness	Experimental	3.22 ± 8.12	2.39 ± 17.10	0.001
	Control	3.12 ± 8.25	3.15 ± 8.32	0.257
	Independent-sample T-test	0.202	0.001	
Attitude	Experimental	4.48 ± 28.19	5.16 ± 62.20	0.001
	Control	4.39 ± 27.55	4.13 ± 29.02	0.208
	Independent-sample T-test	0.237	0.001	
Perceived behavioral control	Experimental	4.93 ± 25.26	5.52 ± 60.18	0.001
	Control	4.77 ± 26.19	4.21 ± 29.06	0.218
	Independent-sample T-test	0.242	0.001	
Subjective norms	Experimental	20.14 ± 4.15	4.86 ± 40.19	0.001
	Control	22.01 ± 4.04	24.25 ± 4.11	0.24
	Independent-sample T-test	0.233	0.001	
physical activity behavioral intention	Experimental	18.18 ± 4.10	42.39 ± 4.50	0.001
	Control	19.06 ± 4.03	21.03 ± 4.09	0.235
	Independent-sample T-test	0.228	0.001	
Nutritional behavioral intention	Experimental	19.80 ± 4.28	41.88 ± 4.35	0.001
	Control	22.14 ± 4.83	20.86 ± 4.94	0.231
	Independent-sample T-test	0.218	0.001	
Physical activity performance	Experimental	7.12 ± 2.25	18.08 ± 1.81	0.001
	Control	8.30 ± 2.18	9.95 ± 2.16	0.197
	Independent-sample T-test	0.253	0.001	
Nutritional performance	Experimental	7.01 ± 2.04	17.23 ± 2.14	0.001
	Control	7.22 ± 2.10	8.13 ± 2.15	0.217
	Independent-sample T-test	0.279	0.001	

Table 4 shows that before the educational intervention, there was no significant difference between the women in the experimental and control groups in terms of weight and BMI, but 6 months after the intervention, a significant difference was found in the experimental group, showing the effect of the education on weight and BMI. However, no significant difference was observed in the control group.

**Table 4:** Comparison of mean and standard deviation of weight and BMI changes in the control group

Variable	Group	Pre-intervention	Six months after intervention	p-value
Body Mass Index (BMI)	Experimental	29.14 ± 3.12	27.12 ± 3.18	0.021
	Control	29.17 ± 3.36	29.56 ± 3.40	0.179
	Independent-sample T-test	0.172	0.042	
Weight	Experimental	77.04 ± 8.39	73.88 ± 10.58	0.001
	Control	78.87 ± 8.83	77.49 ± 8.46	0.156
	Independent-sample T-test	0.148	0.001	

## Discussion

In the present study, the weights and BMIs of the participants in the experimental group were significantly different from the control group six months after the intervention, showing the effectiveness of applying the theory of planning behavior in weight control and improving nutritional and physical activity behaviors. Consistent with this research, educational interventions on nutrition and physical activity in the study by Javadi et al. Led to weight control in the experimental group after two months, showing a significant difference with the control group [33]. In addition, other studies conducted based on nutritional and physical activity interventions reported weight changes in the experimental group as their most important finding, which is in line with the present study [33, 34]. The cross-sectional research by Mazloomy Mahmoodabad et al. showed that all constructs of the TPB were strong predictors of the weight loss behavior [34].

Several factors affect nutrition behaviors and physical activity to control people's weight [35]. The results of the present study showed that six months after the educational intervention, a significant difference was observed in the mean scores of awareness, attitude, subjective norms, perceived behavioral control, nutritional behavioral intention, and nutritional performance in the experimental group. However, none of these constructs showed a significant difference in the control group. Numerous studies have also shown the usefulness of the TPB as a suitable theoretical model to explain the factors determining overweight and obesity control, and have introduced an appropriate framework for designing and implementing the interventions related to it [34, 36]. However, in the study by Pooreh et al., all of the constructs of the TPB in physical activity and nutritional behaviors except for subjective norms in physical activity had a significant increase in the experimental group after the intervention [37]. The difference was probably due to the different intervention programs and the involvement of other important people in the educational and intervention programs used in the two studies, and it could even be said that subjective norms were affected by cultural and social structures of different populations, and this might be the reason for the difference. But the impact of subjective norms on improving nutrition behaviors and physical activity was so important that even other studies that had not used the TPB had examined the impact of other important people, such as parents, on improving health behaviors, and introduced it as important [38, 39].

The results of this study are consistent with those of the study by Etezadi et al. [40]. However, the results of Dehdari et al.'s study showed that attitude towards behavior was not able to predict behavioral intention and nutritional performance, and it was better to focus on subjective norms and perceived behavioral control to improve nutritional intention and performance [41]. Consistent with our study, the results of other studies showed that attitudes toward behavior alongside other constructs were considered as strong predictors [12, 42, 43]. Even the study by Shafieinia et al. indicated that after the intervention, the experimental group had a more positive attitude than the control group to do physical activity and it was stated that positive attitudes had a significant effect on the participation and performance of the experimental group [44].

In their study, Shakerinejad et al. did not examine the participants' awareness, but the results in terms of other constructs of the TPB, including attitude, subjective norms, perceived behavioral control, physical activity behavioral intention, and physical activity performance were in line with the present study [25]. In addition, the results of another study in which the awareness of the participants was not addressed showed that the educational intervention based on the TPB led to significant changes in the mean scores of attitude, perceived behavioral control, physical activity behavioral intention, and physical activity performance in the experimental group after the educational intervention, but subjective norms did not change [44]. Furthermore, the study of Karimzadeh Shirazi et al. showed that subjective norms had a minor role in predicting behavior [45]. However, the study of Mazloomy Mahmoodabad et al. suggested that subjective norms were the most important predictors of obesity and overweight control behaviors [34]. The difference might be due to cultural and social differences in the study groups.

The present study considered the awareness component because the researchers believed that the presence or promotion of awareness in the target group could be one of the effective components in improving health behaviors. In line with our research, some other studies examined the effect of awareness in their educational interventions and showed a significant increase in the mean scores of awareness in the experimental group [46, 47]. However, it could be said that the differences observed in the results of other studies might be due to applying different research methods as well as intervention methods and educational programs, differences in the study populations, and even the differences in the cultural and social status of the target groups. Such differences could affect the individuals’ participation in programs as well as their performance.

## Conclusion

The results of this study showed that designing and implementing effective interventions based on the patterns and theories of health education and health promotion was a very important and decisive issue in improving the health status of the community. Educational and intervention programs that seek success should be designed and implemented through the identification of the components affecting behavior change and in accordance with them. This study showed that education based on the TPB improved the nutritional performance and physical activity of the study population and was able to reduce their weight and BMI. Therefore, considering the effective role of the constructs of this theory in improving nutritional function and physical activity and reducing weight and BMI of overweight and obese women, this theory can be used as a suitable framework in larger populations to improve their health status.

## Data Availability

The datasets used and/or analysed during the current study are available from the corresponding author upon reasonable request.

## Abbreviations

BMI: Body Mass Index
NCDs: Non-Communicable Diseases
TPB: Theory of Planned Behavior
